# Longitudinal Effects of Serum Calcium and Phosphate Levels and Their Ratio on Incident Ischemic Heart Disease among Korean Adults

**DOI:** 10.3390/biom12010103

**Published:** 2022-01-08

**Authors:** Dong Hyuk Jung, Byoungjin Park, Yong Jae Lee

**Affiliations:** 1 Department of Health Check-Up, Yongin Severance Hospital, Yongin-si 16995, Korea; balsan2@yuhs.ac (D.H.J.); bjpark96@yuhs.ac (B.P.); 2 Department of Family Medicine, Gangnam Severance Hospital, 211 Eonju-ro, Seoul 06273, Korea

**Keywords:** serum calcium, serum phosphate, calcium-to-phosphate ratio, cohort study, incident ischemic heart disease

## Abstract

Serum calcium and phosphate levels are controlled by a regulatory system, but their individual concentration tendencies and interactions may affect long-term vascular health. This study aimed to assess the effects of serum calcium and phosphate levels on incident ischemic heart disease (IHD) in a large-scale community-dwelling Korean cohort. We evaluated 15,259 non-diabetic individuals (median age, 45 years; range, 30–85) without previous IHD or ischemic stroke using the Korean National Health Insurance data. The study population was classified based on the calcium, phosphate, and calcium/phosphate ratios. Using Cox proportional hazards regression models, we estimated hazard ratios (HRs) with 95% confidence intervals (CIs) for IHD over 50 months after baseline enrolment. The age- and sex-adjusted incidence of IHD gradually increased with serum calcium and phosphate quartiles and decreased with calcium/phosphate ratio quartiles, with an overall crude rate of 2.1% (315/15,259). After setting the lowest calcium, phosphate, and calcium/phosphate ratio quartiles as a reference group, the HRs (95% CIs) of the highest calcium, phosphate, and calcium/phosphate ratio quartiles for IHD were 1.77 (1.15–2.72), 1.73 (1.18–2.55), and 0.58 (0.39–0.87), respectively, after adjusting for potential confounding variables. Serum calcium and phosphate levels were positively associated with IHD incidence, while the serum calcium/phosphate ratio exhibited an inverse relationship. Serum calcium and phosphate homeostasis may merit serious consideration to understand the pathogenesis of coronary atherosclerosis as a risk modifier for IHD.

## 1. Introduction

The relationship between minerals and cardiovascular disease (CVD) has increasingly important clinical implications. In particular, it is worth noting that calcium, phosphate, and their interactions may be causative factors and biomarkers for CVDs, providing another therapeutic option.

As a ubiquitous divalent cation in the body, calcium plays a role in blood coagulation, muscle contraction, and bone mineralization [1]. Serum calcium levels are mainly controlled by the balance of calcitonin and parathyroid hormone (PTH), which may be influenced by lifestyle factors [2]. In addition, calcium supplementation can be beneficial for bone health in children, older adults, and postmenopausal women [3]. However, there is also increasing concern regarding the possible link between serum calcium levels and cardiovascular events [4,5].

Phosphorus, the second most abundant mineral, is physiologically crucial for bone health, vascular function, energy production, and intracellular signaling [6]. In patients with chronic kidney disease, hyperphosphatemia is recognized as a significant cause of CVDs such as hypertension, atherosclerosis, and coronary or valvular calcification [7,8,9]. Recent data from the general population indicate that higher circulating phosphate levels within the normal range are associated with CVD morbidity and mortality [10].

The regulatory system in blood maintains calcium and phosphate levels under strict control through intestinal absorption, skeletal flux, and renal excretion, but various factors such as aging, dietary pattern, and daily activity may affect serum calcium and phosphate concentrations and their interactions over the course of one’s life [2,11,12,13]. This study aimed to investigate the longitudinal effects of serum calcium and phosphate levels and their ratio on incident ischemic heart disease (IHD) in a large-scale community-dwelling Korean cohort using National Health Insurance Service data.

## 2. Materials and Methods

### 2.1. Study Participants

**Figure 1:**
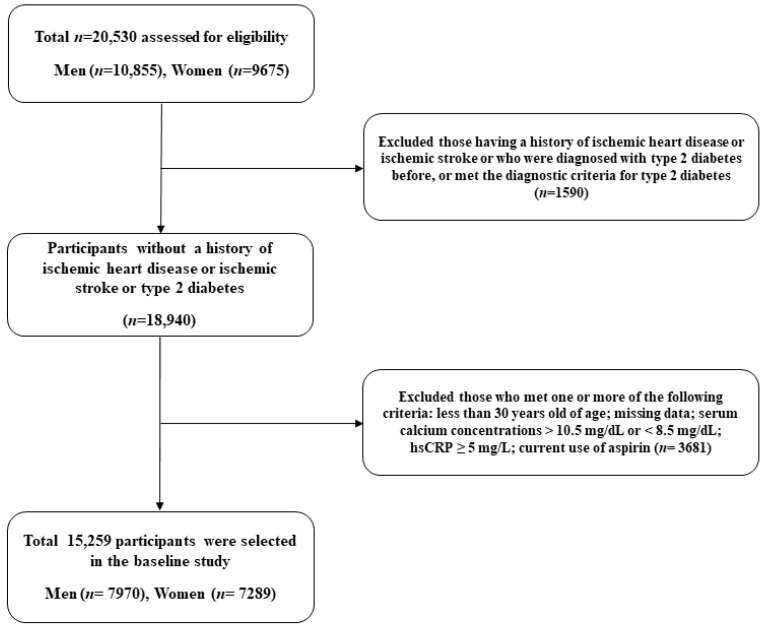
Flowchart for the selection of study participants.

The present research is based on the Health Risk Assessment Study (HERAS) and Korea Health Insurance Review and Assessment Service (HIRA) datasets, which aimed to explore and characterize cardiovascular risk factors among Koreans without diabetes [14,15]. The cohort consisted of 20,530 subjects who voluntarily underwent medical examinations at Yonsei University Gangnam Severance Hospital between November 2006 and June 2010 (Figure 1). We excluded 1590 participants who had previously been diagnosed with IHD or ischemic stroke, a previous diagnosis of type 2 diabetes, or a fasting plasma glucose level ≥ 126 mg/dL. In addition, patients who met at least one of the following criteria were excluded: age < 30 years, missing data, current use of aspirin, serum calcium concentration greater than 10.5 mg/dL or less than 8.5 mg/dL (to rule out calcium homeostasis disorders), or high-sensitivity C-reactive protein (hsCRP) concentration greater than 5.0 mg/L (to rule out acute inflammatory disorders) (*n* = 3681). Consequently, 15,259 individuals (7970 men and 7289 women) were included in the final analysis. This study was conducted according to the ethical principles of the Declaration of Helsinki and was approved by the institutional review board of Yonsei University Gangnam Severance Hospital, Seoul, Korea.

### 2.2. Data Collection

Each participant completed a questionnaire describing their health behaviors, medical history, and any ongoing treatments for the disease. The smoking status was classified as never smoker, ex-smoker, or current smoker. A regular alcohol drinker was defined as a person who consumed more than 140 g of alcohol per week. Regular exercise was defined as moderate physical activity three or more times per week. Bodyweight and height were measured in light indoor clothing and no shoes to the nearest 0.1 kg and 0.1 cm, respectively. The body mass index (BMI) was calculated as weight divided by height squared (kg/m^2^). Systolic blood pressure (SBP) and diastolic blood pressure (DBP) were measured using a standard mercury sphygmomanometer (Baumanometer, W.A. Baum Co Inc., Copiague, NY, USA), with arms supported at the heart level in the sitting position after 10 min of rest. After 12 h overnight fasting, blood samples were collected from the antecubital vein. Total calcium and inorganic phosphate were measured using a Hitachi 7600-110 Chemistry Autoanalyzer (Hitachi, Tokyo, Japan). Hypertension was defined as SBP ≥ 140 mmHg, DBP ≥ 90 mmHg, or current hypertension medication use. The metabolic score for insulin resistance (METS-IR), a non-insulin-based marker for cardiometabolic risk assessment, was calculated as follows: ln ((2× fasting plasma glucose) + triglyceride) × BMI)/ (ln (HDL cholesterol)) [16]. The estimated glomerular filtration rate (eGFR) was calculated using the Modification of Diet in Renal Disease (MDRD) study formula: 186.3 × (serum creatinine -1.154) × (age-0.203) × 0.742 (if female). Chronic kidney disease (CKD) was defined by an eGFR value < 60 mL/min/1.73 m^2^ and/or proteinuria [17].

### 2.3. Exposures and Outcomes

The primary outcome was IHD, which consisted of angina pectoris diagnosed based on ICD-10 code I20 or acute myocardial infarction based on ICD-10 code I21 that occurred after enrolment in the study. To define baseline and study outcomes, we linked a personal 13-digit identification number assigned to each participant by the HIRA between 1 November 2006, and 31 December 2010.

### 2.4. Statistical Analysis

Serum calcium levels were categorized into quartiles as follows: Q1, 8.50–8.90 mg/dL; Q2, 8.91–9.10 mg/dL; Q3, 9.11–9.40 mg/dL; and Q4, 9.41–10.50 mg/dL. Serum phosphate levels were categorized into quartiles as follows: Q1, ≤ 3.40 mg/dL; Q2, 3.41–3.70 mg/dL; Q3, 3.71–4.40 mg/dL; and Q4, ≥ 4.41 mg/dL. Serum calcium-to-phosphate ratios were categorized into quartiles as follows: Q1, ≤ 2.07; Q2, 2.08–2.46; Q3, 2.47–2.73; and Q4, ≥ 2.74. All data are presented as mean ± standard deviation or percentage. The baseline characteristics of the study population were compared according to the serum calcium and phosphate quartiles using an analysis of variance (ANOVA) model for continuous variables and Pearson’s chi-squared test for categorical variables. Using Kaplan–Meier curves, we assessed the cumulative incidence of IHD with the log-rank test to determine whether the distribution of cumulative IHD incidence differed among the groups. In the multivariate analysis, after setting the lowest calcium value quartile as a reference group, hazard ratios (HRs) and 95% confidence intervals (CIs) for incident IHD were calculated using the Cox proportional hazards regression model after adjusting for potential confounding variables. Similarly, we performed a statistical analysis for serum phosphate and calcium-to-phosphate ratio. Furthermore, we evaluated the correlation of calcium, phosphate, and their ratio with increasing age and survival curves after adjusting for age and sex. All analyses were performed using SAS version 9.4 software (SAS Institute Inc., Cary, NC, USA). Statistical significance was determined at *p* < 0.05, with all tests being two-sided.

## 3. Results

### 3.1. Demographic and Biochemical Characteristics

Table 1 and Table 2 show the baseline characteristics of the study population (*n* = 15,259; 7970 men and 7289 women) based on the serum calcium and phosphate quartiles. The mean age, BMI, and serum calcium and phosphate levels were 46.0 ± 9.5 years, 23.4 ± 3.0 kg/m^2^, and 9.14 ± 0.35 and 4.03 ± 0.98 mg/dl, respectively. The mean age was highest in the group with the lowest calcium and highest phosphate quartiles.

The group with the highest calcium quartile showed the highest mean values of BMI, mean arterial pressure, fasting plasma glucose, total cholesterol, triglycerides, and METS-IR. However, the lowest phosphate quartile group exhibited the highest BMI, mean arterial pressure, fasting plasma glucose, triglycerides, and METS-IR. The total cholesterol levels were highest in the highest phosphate quartile group. The prevalence of hypertension was 21.0%, which gradually increased according to serum calcium quartiles. There was no difference in chronic kidney disease among serum calcium quartiles; however, the highest phosphate quartile showed the highest proportion of chronic kidney disease. 

### 3.2. Cumulative Probability for Ischemic Heart Disease

**Figure 2:**
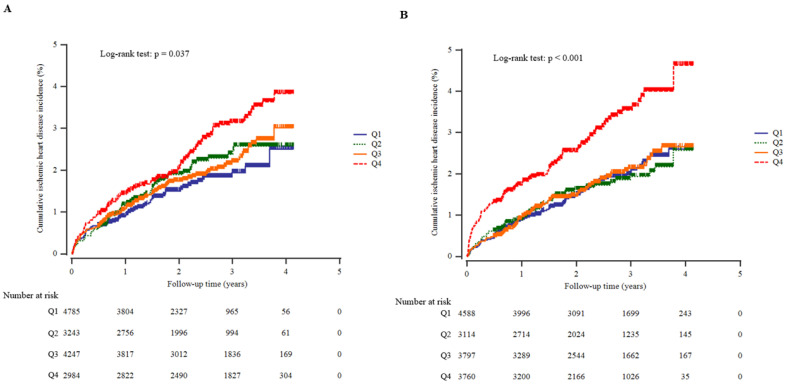
The cumulative probability of ischemic heart disease according to serum calcium quartiles (**A**) and phosphate quartiles (**B**) using Kaplan–Meier plots.

**Figure 3:**
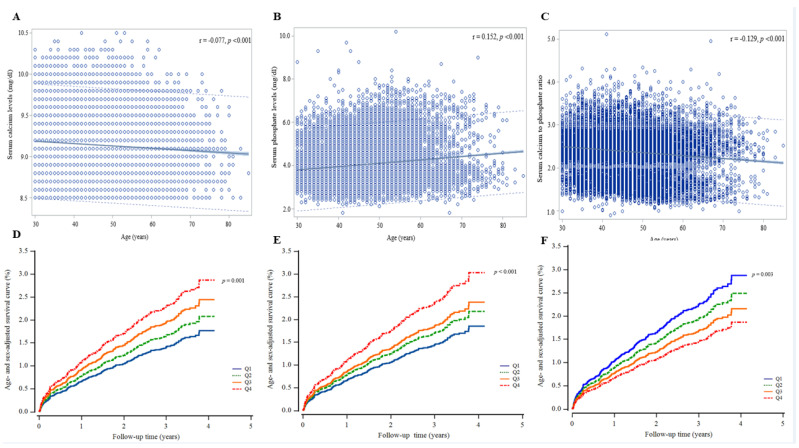
Trends in serum mineral levels according to age and Cox regression survival curves; serum calcium (**A**, **D**), phosphate (**B**, **E**), and calcium-to-phosphate ratio (**C**, **F**).

During the follow-up period, 315 (2.1%, 315/15,259) individuals developed IHD. The IHD incidence rate (per 1000 person-years) was highest with the highest calcium quartile and the highest phosphate quartile. In addition, the highest calcium quartile and the highest phosphate quartile showed a significantly increased cumulative IHD incidence over 50 months following the baseline survey (log-rank test, *p* = 0.037 and *p* < 0.001, respectively) (Figure 2). Serum calcium levels and the calcium-to-phosphate ratio were inversely correlated with increasing age, while serum phosphate levels tended to increase with age (r = −0.077, −0.129, and 0.152, respectively; *p* <0.001 for all). The age- and sex-adjusted Cox regression survival curves show that the elevated calcium and phosphate quartiles and the decreased calcium-to-phosphate ratio quartile group exhibited a higher cumulative incidence of IHD over 50 months (Figure 3).

### 3.3. Multivariate Hazard Ratios for Ischemic Heart Disease

Table 3, Table 4 and Table 5 show the multivariate Cox proportional hazards regression analysis results for predicting IHD based on the serum calcium and phosphate quartiles and their ratio quartiles. Compared with the reference calcium quartile, the HR of incident IHD for the fourth quartile increased after adjusting for potential confounding variables (HR = 1.77; 95% CI, 1.15–2.72). Similarly, individuals in the highest phosphate quartile group had an increased risk of IHD compared with the control group (HR = 1.73; 95% CI, 1.18–2.55). Meanwhile, the HRs for IHD decreased proportionally as the serum calcium-to-phosphate ratio increased. The HRs of incident IHD were 0.74 (95% CI, 0.50–1.11), 0.64 (95% CI, 0.42–0.97), and 0.58 (95% CI, 0.39–0.87) for the second, third, and fourth quartiles, respectively, after adjusting for age, sex, BMI, smoking status, alcohol intake, physical activity, mean arterial blood pressure, hsCRP, chronic kidney disease, serum potassium, and eGFR.

## 4. Discussion

This population-based cohort study among Koreans showed that borderline high serum calcium and phosphate levels are positively associated with IHD incidence, independent of demographic factors, health behaviors, and chronic inflammation. Additionally, individuals with a high serum calcium-to-phosphate ratio exhibited inverse trends in the development of IHD. As age increased, the serum calcium concentration tended to decrease, and serum phosphate appeared to accumulate. Both serum calcium and phosphate seem to be associated with increased IHD incidence even after adjusting for age, but serum phosphate likely plays a more critical role.

There is an ongoing issue regarding the possible relationship between calcium supplements and increased CVD risk [18,19,20]. Taking calcium supplements in addition to proper calcium-containing foods has been shown to contribute to the progression of vascular disease [21]. In addition, an epidemiological data analysis reported that coronary artery disease mortality was 1.13% for each increase in the standard deviation (SD) of the calcium concentration in blood [22]. Similarly, a longitudinal study showed that cerebrovascular events increased 1.37-fold over 12.6 years for every SD increment in serum calcium level. Controversy remains as to whether the risk of calcium supplements is high because Koreans consume less calcium from food than Westerners do. However, recent prospective data among Koreans reported that higher calcium supplementation for ≥12 months without vitamin D was also associated with increased non-fatal myocardial infarction [23]. This may be consistent with the results of this study in that the higher the calcium concentration is over a long period, the higher the risk of cardiovascular disease is.

Serum phosphate levels are closely related to the decline in kidney function with age, and the subsequent risk of CVD is well known [24,25]. Higher serum phosphate levels can contribute to cardiovascular calcification, comparable to traditional cardiovascular risk factors, independent of kidney function [26]. In a study of 3015 young participants aged 18–30 years, higher phosphate levels were found to be a possible risk factor for coronary arterial calcification, even within the normal range [27]. Recent epidemiological studies have also reported that serum phosphate levels in adults—even those with normal kidney function—were significantly associated with CVD events and subclinical coronary arterial calcification [10,28,29]. According to the 2017 Korean National Health and Nutrition Examination Survey (KNHANES), the percentage of adults who consumed less than the daily requirement for calcium was 67.8%, the lowest intake compared to the recommended intake. On the other hand, phosphate was consumed at over 158.6% of the average daily requirement, and this nutritional status was the same regardless of age or sex [30].

There are some possible explanations for the observed associations. Calcium–phosphate homeostasis has emerged as a crucial factor that should be considered in the pathophysiology of CVDs. Increased serum calcium levels could induce alterations in blood coagulation mechanisms and lead to endothelial dysfunction, binding to calcium-sensing receptors, or interaction with pyrophosphates, which are essential inhibitors of tissue calcification; higher serum calcium levels also result in smaller concentrations of serum pyrophosphates and more significant tissue calcification [18,19]. Alterations in gene expression may be induced by prolonged exposure to increased circulating calcium in vascular smooth muscle cells, leading to mutations in calcium-sensing receptors with subsequent enhanced cardiovascular risk [31]. In addition, the higher the intracellular free calcium level is, a secondary messenger system, the greater the catecholamine secretion, vasoconstrictor tone, and arterial blood pressure are [32].

Higher serum phosphate levels may increase the breakdown of the extracellular matrix and induce osteogenic changes in vascular smooth muscle cells [33]. Increased serum phosphate levels can increase reactive oxidative stress and damage endothelial cells [34,35]. In addition, the activated renin-angiotensin-aldosterone system can induce high blood pressure as endothelin-1 production increases via genetic upregulation in the aorta [36,37]. Increased serum phosphate can lead to heart and vascular diseases through various molecular changes resulting from cellular calcium homeostasis. Fibroblast growth factor-23 (FGF-23) may increase, and fetuin-A and α-klotho may become insufficient, resulting in vascular calcification [38,39]. In healthy individuals, the same regulatory system manages calcium and phosphate homeostasis, trying to maintain the physiological level of calcium phosphate products (CPPs) [40]. However, increased CPPs cause coronary arterial disease [28]. In Koreans, the phosphate intake is more than twice as high as the calcium intake, which may contribute to the risk of CVDs due to excessive phosphate levels [30].

Several strengths and limitations require careful consideration, which may affect the interpretation of the study results. A significant strength of our study is that it is a large-scale longitudinal study linked to HIRA data based on the universal coverage system in Korea. Additionally, calcium and phosphate could affect endothelial function, but the possibility of a reciprocal relationship between the two should be considered, and it should be further investigated whether their ratio is associated with IHD. Regarding the study’s limitations, the study participants were volunteers visiting for health promotion screenings in a single hospital and appeared to be slightly healthier than most community-based cohorts, indicating that the study population may not represent the general population. Furthermore, we did not consider genetic susceptibility factors in calcium and phosphate metabolism or the effects of vitamin D and PTH. Lastly, the HERAS-HIRA dataset assessed only newly developed IHD, not coronary angiography or calcium score data.

## 5. Conclusions

In conclusion, borderline high serum calcium and phosphate levels were positively associated with IHD incidence, while a high serum calcium-to-phosphate ratio exhibited an inverse relationship with the development of IHD. Both serum calcium and phosphate seem to be associated with risk of incident IHD, but serum phosphate likely plays a more critical role in Koreans. Serum calcium and phosphate homeostasis may merit serious consideration to understand the pathogenesis of coronary arterial calcification as a risk modifier for IHD.

## Figures and Tables

**Table 1:** Baseline characteristics of the study population according to serum calcium quartiles.

	Quartile of Serum Calcium					
	Q1 *n* = 4785	Q2 *n* = 3243	Q3 *n* = 4247	Q4 *n* = 2984	*p*-Value ^1^	Post hoc ^2^
Serum calcium (mg/dl)	≤8.90	8.91–9.10	9.11–9.40	≥9.41		
Age (years)	46.9 ± 9.5	46.1 ± 9.5	45.7 ± 9.4	45.0 ± 9.4	<0.001	a,b,c,e,f
Male sex (%)	39.2	50.0	58.1	67.3	<0.001	-
Body mass index (kg/m^2^)	23.0 ± 2.9	23.2 ± 2.9	23.5 ± 2.9	24.0 ± 3.1	<0.001	a,b,c,d,e,f
Systolic blood pressure (mmHg)	119.3 ± 15.2	121.3 ± 15.5	123.2 ± 14.9	126.4 ± 15.6	<0.001	a,b,c,d,e,f
Diastolic blood pressure (mmHg)	74.5 ± 9.9	75.9 ± 10.1	77.1 ± 9.7	79.1 ± 10.0	<0.001	a,b,c,d,e,f
Fasting plasma glucose (mg/dl)	90.7 ± 9.7	91.2 ± 9.7	91.7 ± 9.8	92.6 ± 9.8	<0.001	b,c,e,f
Total cholesterol (mg/dl)	186.3 ± 32.3	188.4 ± 31.9	192.5 ± 33.9	197.7 ± 34.4	<0.001	a,b,c,d,e,f
Triglyceride (mg/dl)	109.7 ± 66.7	119.0 ± 77.8	129.7 ± 83.3	154.3 ± 116.3	<0.001	a,b,c,d,e,f
HDL cholesterol (mg/dl)	53.7 ± 12.9	53.4 ± 12.5	53.5 ± 12.5	52.9 ± 12.6	0.078	
C-reactive protein (mg/L)	0.9 ± 0.9	0.8 ± 0.9	0.9 ± 0.9	0.9 ± 0.9	<0.001	e
METS-IR	33.1 ± 6.1	33.7 ± 6.2	34.4 ± 6.2	35.6 ± 6.7	<0.001	a,b,c,d,e,f
Serum phosphate (mg/dl)	4.3 ± 1.2	4.0 ± 0.9	3.9 ± 0.8	3.9 ± 0.8	<0.001	a,b,c,d,e
Serum potassium (mmol/l)	3.9 ± 0.4	4.0 ± 0.3	4.1 ± 0.3	4.2 ± 0.4	<0.001	a,b,c,d,e,f
eGFR (mL/min/1.73 m^2^)	83.5 ± 13.5	83.6 ± 13.2	82.9 ± 13.4	81.8 ± 12.7	<0.001	c,e,f
Current smoker (%)	19.2	22.1	27.7	32.8	<0.001	-
Alcohol drinking (%)	35.2	42.8	47.8	53.6	<0.001	-
Regular exercise (%)	31.1	29.9	31.2	31.9	0.378	-
Hypertension (%)	16.9	19.1	22.1	28.1	<0.001	-
Chronic kidney disease (%)	2.2	1.9	2.0	2.3	0.622	-

^1^ *p*-values were calculated using one-way ANOVA or Pearson’s chi-squared test. ^2^ Post hoc analysis with the Bonferroni method: a, Q1 versus Q2; b, Q1 versus Q3; c, Q1 versus Q4; d, Q2 versus Q3; e, Q2 versus Q4; f, Q3 versus Q4.

**Table 2:** Baseline characteristics of the study population according to serum phosphate quartiles.

	Quartile of Serum Phosphate					
	Q1 *n* = 4588	Q2 *n* = 3114	Q3 *n* = 3797	Q4 *n* = 3760	*p*-Value ^1^	Post hoc ^2^
Serum phosphate (mg/dl)	≤3.40	3.41–3.70	3.71–4.40	≥4.41		
Age (years)	45.6 ± 9.4	44.5 ± 9.1	45.5 ± 9.6	48.4 ± 9.4	<0.001	a,b,d,e,f
Male sex (%)	37.9	46.7	38.0	54.7	<0.001	-
Body mass index (kg/m^2^)	23.9 ± 2.9	23.3 ± 3.0	23.1 ± 3.0	23.1 ± 2.9	<0.001	a,b,c,d,e
Systolic blood pressure (mmHg)	125.2 ± 15.8	121.3 ± 15.1	120.4 ± 15.7	121.2 ± 14.6	<0.001	a,b,c
Diastolic blood pressure (mmHg)	78.2 ± 10.3	75.7 ± 9.9	75.1 ± 10.2	76.1 ± 9.4	<0.001	a,b,c,f
Fasting plasma glucose (mg/dl)	92.3 ± 9.5	90.6 ± 9.0	90.4 ± 9.5	92.3 ± 10.6	<0.001	a,b,e,f
Total cholesterol (mg/dl)	185.7 ± 31.2	187.0 ± 32.2	190.5 ± 34.1	200.0 ± 34.3	<0.001	b,c,d,e,f
Triglyceride (mg/dl)	134.6 ± 88.9	122.0 ± 80.2	121.7 ± 97.7	123.1 ± 75.9	<0.001	a,b,c
HDL cholesterol (mg/dl)	51.7 ± 12.0	54.1 ± 12.6	54.9 ± 12.7	53.5 ± 13.2	<0.001	a,b,c,d,f
C-reactive protein (mg/L)	0.9 ± 0.9	0.8 ± 0.9	0.9 ± 0.9	0.9 ± 0.9	<0.001	e
METS-IR	35.2 ± 6.2	33.8 ± 6.3	33.3 ± 6.3	33.7 ± 6.3	<0.001	a,b,c,d,f
Serum calcium (mg/dl)	9.2 ± 0.3	9.2 ± 0.3	9.2 ± 0.3	9.0 ± 0.3	<0.001	a,b,c,d,e,f
Serum potassium (mmol/l)	4.1 ± 0.3	4.1 ± 0.3	4.1 ± 0.3	3.7 ± 0.4	<0.001	c,e,f
eGFR (mL/min/1.73 m^2^)	84.1 ± 13.0	84.7 ± 13.0	84.0 ± 13.7	79.4 ± 12.7	<0.001	c,e,f
Current smoker (%)	26.8	23.0	22.7	26.2	<0.001	-
Alcohol drinking (%)	51.5	44.9	40.0	37.8	<0.001	-
Regular exercise (%)	30.8	29.1	29.4	34.6	<0.001	-
Hypertension (%)	24.9	18.5	18.4	20.9	<0.001	-
Chronic kidney disease (%)	1.9	1.3	1.5	3.6	0.011	-

^1^ *p*-values were calculated using one-way ANOVA or Pearson’s chi-squared test. ^2^ Post hoc analysis with the Bonferroni method: a, Q1 versus Q2; b, Q1 versus Q3; c, Q1 versus Q4; d, Q2 versus Q3; e, Q2 versus Q4; f, Q3 versus Q4.

**Table 3:** Hazard ratios and 95% confidence intervals for new-onset ischemic heart disease (IHD) according to serum calcium quartiles.

	Serum Calcium Quartiles				
	Q1	Q2	Q3	Q4	*p*-Value
New cases of IHD, n	71	64	88	92	
Mean follow-up, years	2.0 ± 1.0	2.3 ± 1.0	2.6 ± 1.0	3.0 ± 1.0	
Person-years of follow-up	9680	7461	10,938	8822	
Incidence rate/1000 person-years	7.3	8.6	8.0	10.4	
Model 1	1.00 (reference)	1.24 (0.88–1.73)	1.22 (0.89–1.67)	1.72 (1.25–2.36)	0.007
Model 2	1.00 (reference)	1.30 (0.91–1.86)	1.32 (0.95–1.84)	1.68 (1.20–2.36)	0.029
Model 3	1.00 (reference)	1.43 (0.97–2.12)	1.42 (0.96–2.10)	1.77 (1.15–2.72)	0.066

Model 1: Adjusted for age and sex. Model 2: Adjusted for age, sex, body mass index, smoking status, alcohol intake, and physical activity. Model 3: Adjusted for age, sex, body mass index, smoking status, alcohol intake, physical activity, mean arterial blood pressure, C-reactive protein level, chronic kidney disease, serum phosphate, serum potassium, and eGFR.

**Table 4:** Hazard ratios and 95% confidence intervals for new-onset ischemic heart disease (IHD) according to serum phosphate quartiles.

	Serum Phosphate Quartiles				
	Q1	Q2	Q3	Q4	*p*-Value
New cases of IHD, n	82	53	71	109	
Mean follow-up, years	2.5 ± 1.1	2.5 ± 1.1	2.5 ± 1.1	2.2 ± 1.0	
Person-years of follow-up	11400	7680	9547	8275	
Incidence rate/1000 person-years	7.2	6.9	7.4	13.2	
Model 1	1.00 (reference)	1.18 (0.83–1.67)	1.29 (0.93–1.79)	1.65 (1.23–2.20)	0.007
Model 2	1.00 (reference)	1.06 (0.73–1.54)	1.23 (0.87–1.74)	1.65 (1.22–2.22)	0.006
Model 3	1.00 (reference)	0.96 (0.60–1.54)	1.20 (0.79–1.82)	1.73 (1.18–2.55)	0.018

Model 1: Adjusted for age and sex. Model 2: Adjusted for age, sex, body mass index, smoking status, alcohol intake, and physical activity. Model 3: Adjusted for age, sex, body mass index, smoking status, alcohol intake, physical activity, mean arterial blood pressure, C-reactive protein level, chronic kidney disease, serum calcium, serum potassium, and eGFR.

**Table 5:** Hazard ratios and 95% confidence intervals for new-onset ischemic heart disease (IHD) according to serum calcium-to-phosphate ratio quartiles.

	Serum Calcium-to-Phosphate Ratio Quartiles				
	Q1	Q2	Q3	Q4	*p*-Value
New cases of IHD, n	108	69	63	75	
Mean follow-up, years	2.2 ± 1.0	2.4 ± 1.1	2.5 ± 1.1	2.6 ± 1.0	
Person-years of follow-up	8360	9468	9202	9871	
Incidence rate/1000 person-years	12.9	7.3	6.8	7.6	
Model 1	1.00 (reference)	0.79 (0.58–1.08)	0.71 (0.52–0.97)	0.65 (0.48–0.88)	0.024
Model 2	1.00 (reference)	0.69 (0.49–0.96)	0.68 (0.49–0.94)	0.64 (0.47–0.87)	0.012
Model 3	1.00 (reference)	0.74 (0.50–1.11)	0.64 (0.42–0.97)	0.58 (0.39–0.87)	0.041

Model 1: Adjusted for age and sex. Model 2: Adjusted for age, sex, body mass index, smoking status, alcohol intake, and physical activity. Model 3: Adjusted for age, sex, body mass index, smoking status, alcohol intake, physical activity, mean arterial blood pressure, C-reactive protein level, chronic kidney disease, serum potassium, and eGFR.

## Data Availability

The data underlying this article will be shared upon reasonable request from the corresponding author.

## References

[1] Papadopoulou A., Bountouvi E., Karachaliou F.-E. (2021). The molecular basis of calcium and phosphorus inherited metabolic disorders. Genes.

[2] Lombardi G., Ziemann E., Banfi G., Corbetta S. (2020). Physical activity-dependent regulation of parathyroid hormone and calcium-phosphorous metabolism. Int. J. Mol. Sci..

[3] North American Menopause Society (2006). The role of calcium in peri- and postmenopausal women: 2006 position statement of the north american menopause society. Menopause.

[4] Myung S.-K., Kim H.-B., Lee Y.-J., Choi Y.-J., Oh S.-W. (2021). Calcium supplements and risk of cardiovascular disease: A meta-analysis of clinical trials. Nutrients.

[5] Reid I.R. (2013). Cardiovascular effects of calcium supplements. Nutrients.

[6] Zhou C., Shi Z., Ouyang N., Ruan X. (2021). Hyperphosphatemia and cardiovascular disease. Front. Cell Dev. Biol..

[7] Tsuchiya K., Akihisa T. (2021). The importance of phosphate control in chronic kidney disease. Nutrients.

[8] Kestenbaum B., Sampson J.N., Rudser K.D., Patterson D.J., Seliger S.L., Young B., Sherrard D.J., Andress D.L. (2005). Serum phosphate levels and mortality risk among people with chronic kidney disease. J. Am. Soc. Nephrol..

[9] Lim C.C., Teo B.W., Ong P.G., Cheung C.Y., Lim S.C., Chow K.Y., Meng C.C., Lee J., Tai E.S., Wong T.Y. (2015). Chronic kidney disease, cardiovascular disease and mortality: A prospective cohort study in a multi-ethnic asian population. Eur. J. Prev. Cardiol..

[10] McGovern A.P., de Lusignan S., van Vlymen J., Liyanage H., Tomson C.R., Gallagher H., Rafiq M., Jones S. (2013). Serum phosphate as a risk factor for cardiovascular events in people with and without chronic kidney disease: A large community based cohort study. PLoS ONE.

[11] Cannata-Andía J.B., Carrillo-López N., Messina O.D., Hamdy N.A.T., Panizo S., Ferrari S.L. (2021). Pathophysiology of vascular calcification and bone loss: Linked disorders of ageing?. Nutrients.

[12] Saito Y., Sakuma M., Narishima Y., Yoshida T., Kumagai H., Arai H. (2021). Greater consumption of noodle is associated with higher serum phosphorus levels: A cross-sectional study on healthy participants. J. Clin. Biochem. Nutr..

[13] Gutiérrez O.M., Isakova T., Enfield G., Wolf M. (2011). Impact of poverty on serum phosphate concentrations in the third national health and nutrition examination survey. J. Ren. Nutr..

[14] Kim J.J., Yoon J., Lee Y.-J., Park B., Jung D.-H. (2021). Predictive value of the atherogenic index of plasma (AIP) for the risk of incident ischemic heart disease among non-diabetic koreans. Nutrients.

[15] Lee S.-B., Park B.-J., Lee Y.-J., Jung D.-H. (2021). Early chronic kidney disease (G1-G3a) in combination with steatosis as a predictor of incident ischemic heart disease: A longitudinal study in non-diabetic koreans. Biomedicines.

[16] Yoon J., Jung D., Lee Y., Park B. (2021). The metabolic score for insulin resistance (METS-IR) as a predictor of incident ischemic heart disease: A longitudinal study among korean without diabetes. J. Pers. Med..

[17] Kang H.-T., Linton J.A., Kwon S.K., Park B.-J., Lee J.H. (2016). Ferritin level is positively associated with chronic kidney disease in korean men, based on the 2010–2012 korean national health and nutrition examination survey. Int. J. Environ. Res. Public Health.

[18] Wang L., Manson J.E., Sesso H.D. (2012). Calcium intake and risk of cardiovascular disease: A review of prospective studies and randomized clinical trials. Am. J. Cardiovasc. Drugs.

[19] Reid I.R., Bolland M.J., Avenell A., Grey A. (2011). Cardiovascular effects of calcium supplementation. Osteoporos Int..

[20] Bolland M.J., Grey A., Reid I.R. (2013). Calcium supplements and cardiovascular risk: 5 years on. Ther. Adv. Drug Saf..

[21] Bolland M.J., Grey A., Avenell A., Gamble G.D., Reid I.R. (2011). Calcium supplements with or without vitamin d and risk of cardiovascular events: Reanalysis of the women’s health initiative limited access dataset and meta-analysis. BMJ.

[22] Reid I., Gamble G., Bolland M. (2016). Circulating calcium concentrations, vascular disease and mortality: A systematic review. J. Intern. Med..

[23] Kim K.J., Kim M.S., Hong N., Bae J.H., Kim K.J., Kim N.H., Rhee Y., Lee J., Kim S.G. (2021). Cardiovascular risks associated with calcium supplementation in patients with osteoporosis: A nationwide cohort study. Eur. Heart J. Cardiovasc. Pharmacother..

[24] Reiss A.B., Miyawaki N., Moon J., Kasselman L.J., Voloshyna I., D’Avino R., De Leon J. (2018). Ckd, arterial calcification, atherosclerosis and bone health: Inter-relationships and controversies. Atherosclerosis.

[25] Palmer S.C., Hayen A., Macaskill P., Pellegrini F., Craig J.C., Elder G.J., Strippoli G.F. (2011). Serum levels of phosphorus, parathyroid hormone, and calcium and risks of death and cardiovascular disease in individuals with chronic kidney disease: A systematic review and meta-analysis. JAMA.

[26] Tuttle K.R., Short R.A. (2009). Longitudinal relationships among coronary artery calcification, serum phosphorus, and kidney function. Clin. J. Am. Soc. Nephrol..

[27] Foley R.N., Collins A.J., Herzog C.A., Ishani A., Kalra P.A. (2009). Serum phosphorus levels associate with coronary atherosclerosis in young adults. J. Am. Soc. Nephrol..

[28] Shin S., Kim K.J., Chang H.J., Cho I., Kim Y.J., Choi B.W., Rhee Y., Lim S.K., Yang W.I., Shim C.Y. (2012). Impact of serum calcium and phosphate on coronary atherosclerosis detected by cardiac computed tomography. Eur. Heart J..

[29] Kai R., Murtala B., Zainuddin A.A., Amin M., Ilyas M. (2019). Correlation between serum calcium and phosphate with coronary artery calcium scores on cardiac msct examination. St. Med..

[30] Lee Y.-K., Choi M.-K., Hyun T., Lyu E.-S., Park H., Ro H.-K., Heo Y.-R. (2020). Analysis of dietary calcium and phosphorus intakes and contribution rates of major dish groups according to gender, age, and region in korea. Korean J. Community Nutr..

[31] März W., Seelhorst U., Wellnitz B., Tiran B., Obermayer-Pietsch B., Renner W., Boehm B.O., Ritz E., Hoffmann M.M. (2007). Alanine to serine polymorphism at position 986 of the calcium-sensing receptor associated with coronary heart disease, myocardial infarction, all-cause, and cardiovascular mortality. J. Clin. Endocrinol. Metab..

[32] Resnick L.M. (1993). Ionic basis of hypertension, insulin resistance, vascular disease, and related disorders the mechanism of “Syndrome X”. Am. J. Hypertens..

[33] Lau W.L., Pai A., Moe S.M., Giachelli C.M. (2011). Direct effects of phosphate on vascular cell function. Adv. Chronic Kidney Dis..

[34] Amann K., Törnig J., Kugel B., Gross M.-L., Tyralla K., El-Shakmak A., Szabo A., Ritz E. (2003). Hyperphosphatemia aggravates cardiac fibrosis and microvascular disease in experimental uremia. Kidney Int..

[35] Chue C.D., Townend J.N., Steeds R.P., Ferro C.J. (2010). Republished paper: Arterial stiffness in chronic kidney disease: Causes and consequences. Postgrad. Med. J..

[36] Bozic M., Panizo S., Sevilla M.A., Riera M., Soler M.J., Pascual J., Lopez I., Freixenet M., Fernandez E., Valdivielso J.M. (2014). High phosphate diet increases arterial blood pressure via a parathyroid hormone mediated increase of renin. J. Hypertens..

[37] Olmos G., Martínez-Miguel P., Alcalde-Estevez E., Medrano D., Sosa P., Rodríguez-Mañas L., Naves-Diaz M., Rodríguez-Puyol D., Ruiz-Torres M.P., López-Ongil S. (2017). Hyperphosphatemia induces senescence in human endothelial cells by increasing endothelin-1 production. Aging Cell.

[38] Muñoz-Castañeda J.R., Rodelo-Haad C., Pendon-Ruiz de Mier M.V., Martin-Malo A., Santamaria R., Rodriguez M. (2020). Klotho/fgf23 and wnt signaling as important players in the comorbidities associated with chronic kidney disease. Toxins.

[39] Voelkl J., Lang F., Eckardt K.-U., Amann K., Kuro-o M., Pasch A., Pieske B., Alesutan I. (2019). Signaling pathways involved in vascular smooth muscle cell calcification during hyperphosphatemia. Cell. Mol. Life Sci..

[40] Quinn S.J., Thomsen A.R., Pang J.L., Kantham L., Bräuner-Osborne H., Pollak M., Goltzman D., Brown E.M. (2013). Interactions between calcium and phosphorus in the regulation of the production of fibroblast growth factor 23 in vivo. Am. J. Physiol. Endocrinol. Metab..

